# An Anthocyanin-Rich Mixed-Berry Intervention May Improve Insulin Sensitivity in a Randomized Trial of Overweight and Obese Adults

**DOI:** 10.3390/nu11122876

**Published:** 2019-11-25

**Authors:** Patrick M. Solverson, Theresa R. Henderson, Hawi Debelo, Mario G. Ferruzzi, David J. Baer, Janet A. Novotny

**Affiliations:** 1 USDA, ARS, Beltsville Human Nutrition Research Center, Beltsville, MD 20705, USA; patrick.solverson@uvm.edu (P.M.S.); theresa.henderson@usda.gov (T.R.H.); david.baer@usda.gov (D.J.B.); 2 Department of Nutrition and Food Sciences, University of Maryland, College Park, MD 20742, USA; 3 Department of Nutrition and Food Sciences, University of Vermont, Burlington, VT 05405, USA; 4 Plants for Human Health Institute, North Carolina State University, Kannapolis, NC 28081, USA; hdebelo@ncsu.edu (H.D.); mferruz@ncsu.edu (M.G.F.)

**Keywords:** berries, insulin sensitivity, diabetes, T2DM

## Abstract

Evidence supports the beneficial effects of berries on glucoregulation, possibly related to flavonoid content, fiber content, or both. The purpose of this study was to assess the potential of mixed berries to improve insulin sensitivity and to identify the potential role of flavonoids and fiber. In a randomized cross-over trial with four treatment periods, overweight/obese men and women were fed a controlled 45% fat diet for one week prior to a meal-based glucose tolerance test. The same base diet was provided during each feeding period with the addition of one of four treatments: whole mixed berries, sugar matched mixed berry juice, sugar matched gelatin, and sugar/fiber matched gelatin. Subjects then completed a meal-based oral glucose tolerance test. Serum glucose, insulin and non-esterified fatty acids were not different between individual treatments. However, in a secondary analysis, the combined berry preparations resulted in a lower serum insulin area under the curve (difference of 0.15 ± 0.066 ln pmol min/mL, mean ± SE, *p* = 0.0228), compared to the combined gelatin treatments, while the difference for serum glucose did not quite meet statistical significance (difference of 0.17 ± 0.093 ln mg·min/dL, mean ± SE, *p* = 0.0738). These results suggest the potential for mixed berry preparations to improve post-prandial insulin response.

## 1. Introduction

Improvement of glucoregulation through diet has potential for a significant impact on public health [1]. Diabetes mellitus is a major public health challenge of the twenty-first century [2] and is estimated to be the cause of approximately 1.5 million deaths yearly [1]. Diet is a modifiable risk factor that could reduce the prevalence of and risk for type 2 diabetes [1]. Moreover, it has been estimated that the majority of type 2 diabetes could be prevented by a healthier lifestyle [3]. Berries are a dietary/lifestyle factor that may contribute to a reduction in the development of diabetes [4].

Berry consumption has been investigated for its health-promoting effects, specifically the ability to attenuate the comorbidities of diet-induced obesity. Evidence from previous human intervention studies described the beneficial effects of berries on insulin sensitivity and glucoregulation [5,6,7,8,9,10,11,12,13,14,15,16,17,18,19,20]. These effects have been observed with both longer-term [6,12,20] and acute [7,8,9,10,16,17] consumption of berries or berry extracts, powders, or products. However, other studies have produced null results with respect to aspects of glucoregulation [21,22,23,24,25], and these contrasting results may be related to differing berry compositions or lack of dietary control prior to the glucoregulatory testing, as dietary pattern prior to testing of glucose metabolism can influence results [26,27,28]. Berries are rich in polyphenols, especially anthocyanins, which are thought to influence several mechanisms of glucose metabolism to ultimately improve clinical measures [4]. Berries also contain fiber, which may play a role in the observed glucoregulatory effects of berries [29].

The objective of this study was to investigate the glucoregulatory effects of mixed berries to provide a wide array of anthocyanins under very highly controlled conditions and to identify the potential role of flavonoids/anthocyanins and/or fiber in these potential health benefits. For this study, berries and control foods were provided with a high-fat diet, similarly to preclinical studies demonstrating the preventative effects of berries on the development of adiposity and glucose dysregulation in rodent models [30,31,32,33,34,35,36,37,38].

## 2. Materials and Methods

### 2.1. Study Design

This study was conducted at the U.S. Department of Agriculture’s Beltsville Human Nutrition Research Center in Beltsville, MD, between January 2018 and March 2019 (Clinical Trial Registry NCT03458858). The study was conducted as a randomized crossover with 4 treatment periods. Each treatment period was 8 days, with a 2-week break between treatment periods. During the first 7 days, the subjects consumed a fully controlled base diet with the addition of one of four treatment foods: whole mixed berries, pressed mixed berry juice, sugar matched gelatin, or sugar matched fiber-enriched gelatin. On the 8th day of each treatment period, subjects were administered a meal-based oral glucose tolerance test. Blood samples were collected prior to the test meal and every 30 minutes after the test meal for 3 hours. Serum was analyzed for glucose, insulin, and non-esterified fatty acids.

### 2.2. Subjects

Volunteers were recruited from the geographical area surrounding the Beltsville Human Nutrition Research Center, which is a population-dense area located between Baltimore, Maryland, and Washington, DC. Recruitment was conducted by email to the Beltsville Human Nutrition Center volunteer database (which contains approximately 1500 email address entries), local Federal agencies, and the University of Maryland. Subjects were required to have the following characteristics: between 21 and 75 years old, BMI greater than 25 kg/m^2^, not pregnant/lactating, and non-smoking. In addition, subjects were required to have no history of any of the following: bariatric surgery, gastrointestinal disorder, malabsorption disorder, metabolic disorder, diabetes, certain cancers, or use of blood thinners or other medications or supplements that could interfere with the study outcomes. All the procedures were approved by the Chesapeake Institutional Review Board (Columbia, MD), and all subjects provided written informed consent.

### 2.3. Background Diets

Fully controlled diets were provided to all subjects for the entirety of each 8-day treatment phase, because dietary pattern preceding a glucose tolerance test can affect results [26,27,28]. Menus were comprised of foods characteristic of a typical American diet pattern, including meat, grains, fruits, vegetables, eggs, and dairy. An 8-day rotation of menus was provided and menus/foods were provided in identical order and amount for each subject during each treatment period and prior to each glucose tolerance test. The background diets were devoid of anthocyanins and were designed to be high in fat, providing 45% of energy from fat, 42% of energy from carbohydrate, and 13% of energy from protein, which was equivalent to 0.86 g protein per kg, thus meeting the RDA for protein. The diet was designed to be high in fat based on preclinical studies demonstrating the preventative effects of berries on development of adiposity and glucose dysregulation in rodent models. The energy requirement for each subject was calculated as the average of the Harris–Benedict equation and the Mifflin–St. Jeor equation multiplied by an activity factor which was based on self-reported daily and weekly exercise. Foods were scaled according to overall energy need and all foods were weighed to the nearest gram. Subjects were instructed to consume all the foods provided and only the foods provided. Coffee and tea were limited to 16 ounces daily.

The subjects consumed breakfast at the Beltsville Human Nutrition Research Center Monday through Friday under the supervision of a dietician or diet tech. Lunches, dinners, and weekend meals were packed for carry-out and consumed off-site.

### 2.4. Dietary Treatments

The primary treatment food was whole mixed berries, to provide a wide array of anthocyanins, and the other treatment foods were designed to serve as different types of control foods for the whole berries. The berries were a combination of equal parts blackberries, blueberries, cranberries, raspberries, and strawberries. The berries were consumed daily at breakfast and dinner. Serving size was scaled according to overall energy needs, such that 100 grams of berries were consumed for every 450 kilocalories of overall energy requirement, resulting in a range of 400 g/day for subjects with the lowest energy intake to 800 g/day for subjects with the highest energy intake. Berries were provided in equal amounts, and each type of berry ranged from 80 to 160 grams per day, as scaled with energy requirement. Prior to the start of the intervention, the berries were analyzed for sugar (monosaccharide) and fiber (soluble and insoluble) content, and the sugar and fiber contents were used to plan the comparator foods.

Three comparator foods were included in the study: sugar-sweetened gelatin, sugar-sweetened fiber enriched gelatin, and pressed berry juice. The sugar-sweetened gelatin was created from a recipe of water (91 g/100 g), gelatin powder (1.5 g/100 g), fructose powder (3.5 g/100 g), glucose powder (3.8 g/100 g), and red and blue food coloring (<0.1 g/100 g). The sugar-sweetened gelatin was matched to the berries for glucose, fructose, and mass consumed, and served as a control food devoid of the proposed active ingredients of anthocyanins and fiber. The sugar-sweetened fiber enriched gelatin was made from a recipe of water (89 g/100 g), gelatin powder (1.5 g/100 g), fructose powder (3.5 g/100 g), glucose powder (3.8 g/100 g), cellulose powder (1.8 g/100 g), and red and blue food coloring (<0.1 g/100 g). The sugar-sweetened fiber enriched gelatin was matched to the berries for glucose, fructose, fiber, and mass consumed, and served as a control food containing fiber but devoid of anthocyanins. The juice was pressed from berries in equal portions of each berry by weight from the same lots as those used for the whole berry treatment. The juice was extracted using a manual fruit press, then filtered through a cheese cloth. The juice was matched to the whole berries for sugar, serving as a control food containing phenolic compounds but no fiber.

Berries and berry juice were analyzed for the following: sugar profile according to the method of Mason and Slover [39] and soluble and insoluble fiber by AOAC method 991.43 by Covance Laboratories (Madison, WI, USA).

### 2.5. Identification of Phenolics in Berries and Berry Juice by LC-MS/MS

Profile of phenolic compounds in the mixed berries (after freeze-drying) and in the pressed berry juice was determined using the Waters UPLC Acquity H Class system (Milford, MA, USA) equipped with a QDa and a PDA detector based on previously published analyses with minor modifications [40]. Separation was performed on a BEH C18 column (1.7 µm, 2.1 mm × 100 mm) at a flow rate of 0.5 mL/min. Samples were eluted with a gradient of 0.1% formic acid in acetonitrile (solvent A) and 2% formic acid in water (solvent B) as follows: 0 min, 100% B; 1.0 min, 94% B; 4.0 min, 91% B; 6.0 min, 87% B; 9.0 min, 65% B; 9.8 min, 100% B; 14.5 min, 100% B. Selected Ion Recording (SIR) parameters were optimized using authentic standards for the identification and quantification of target compounds.

### 2.6. Meal-Based Glucose Tolerance Test

A meal-based oral glucose tolerance test was administered on the morning of day 8. Subjects fasted for 12 hours prior to the test and had engaged in no strenuous activity the morning of the test. An indwelling catheter was placed in the antecubital vein, after which 2 baseline blood samples were collected 15 minutes apart (2 collections were averaged to ensure an accurate baseline value). Additional blood samples were collected every 30 minutes for 3 hours after consumption of the challenge meal. Serum was stored at −80 °C until analysis after samples were collected for the study.

The challenge meal for the glucose tolerance test was designed using a newly created recipe to increase glucose load compared to standard recipes using sucrose as the sugar source. A recipe created using sucrose, a disaccharide of glucose and fructose, would have less impact on blood glucose than the same recipe created with glucose. The challenge meal was a pancake, syrup, berry or control treatment food, and an 8-ounce bottle of water. All these foods were consumed together within a 15-minute period between 6:30 and 7:00 AM. The pancake was made from all-purpose flour, egg, buttermilk, and glucose powder. Eighty grams of batter were weighed and pan-cooked for the glucose tolerance test. The pancake was served with a small container of pancake syrup made from 60 g light corn syrup (Karo) and 1.2 g artificial maple flavoring, together providing 68 g of glucose as monosaccharide. Subjects consumed the pancake and syrup with their assigned treatment food. The treatment food serving sizes for the glucose tolerance test were matched to provide equal amounts of sugar and were 300 grams for whole berries, 300 g for gelatin, 300 g for fiber-enriched gelatin, and 288 g for berry juice. The treatment foods provided an additional 11 g of glucose. The total glucose as monosaccharide provided from the meal-based glucose tolerance test was 79 g, which is similar to the standard dose of 75 g for an oral glucose tolerance test. Subjects were instructed to dip the pancake into the syrup, then to scrape the plate and syrup container clean with a small spatula to ensure complete consumption. Subjects also rinsed their treatment food containers with water and scraped sides with a spatula, again to ensure complete consumption.

Serum glucose and nonesterified fatty acids (NEFA) were measured with a clinical chemistry analyzer (Vitros 5,1 Orthoclinical Diagnostics, San Jose, CA, USA) using colorimetric endpoint assays (CV = 1.1% and 2.0% for glucose and NEFA, respectively). Serum insulin was measured by immunoassay in a microfluidic cartridge (Ella, Proteinsimple, Raritan, NJ, USA, CV = 2.1%).

### 2.7. Calculations and Statistics

A power calculation was performed based on data from a previously published study to detect a difference of 2546 mU/L*hour for insulin area under the curve with 80% power based on a previously published study of a similar design [19]. Volunteers were randomly assigned to one of 9 treatment sequences using a random number generator by an investigator. Diet treatments were color-coded for masking and color codes were used by dietitians, investigators, phlebotomists, analysts, and the study statistician. Codes were unmasked after primary statistical analyses were completed. Incremental area under the curve (iAUC) was calculated for glucose and insulin, and area under the curve (AUC) for NEFAs using central Riemann-sum. Data were analyzed by ANCOVA with repeated measures. Prior to ANCOVA, data were evaluated for outliers, normality and heterogeneity of variance, and transformed if needed. Outliers were defined as individuals with glucose or insulin responses >3 standard deviations above the sample mean (suggesting that these individuals had characteristics of diabetes). The model (PROC MIXED, SAS version 9.4, Cary, NC, USA) included covariates for BMI, age, sex, treatment period, and treatment sequence. Subject was included as a random effect. Additionally, for glucose and insulin response to the meal challenge, time was included in the model as a repeated term, as well as the time*treatment interaction. A per protocol, a statistical analysis was performed in which data were included from subjects who completed at least one treatment period. A secondary analysis was performed (after unmasking) to compare the results of the combined berry treatments (whole + juice) to the control treatments. Statistical significance was defined at *p* < 0.05.

## 3. Results

**Figure 1:**
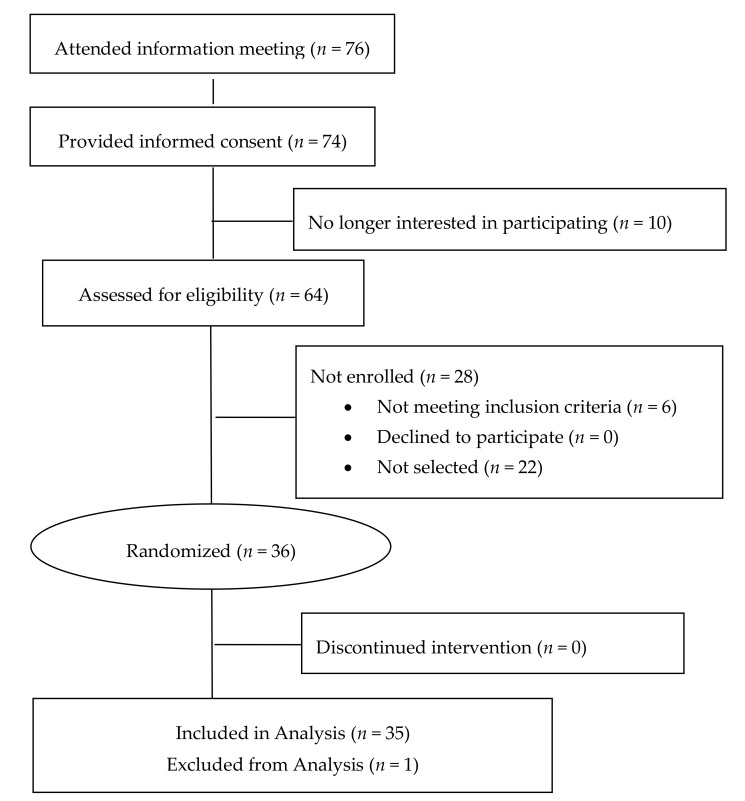
CONSORT (Consolidated Standards of Reporting Trials) diagram. One subject was excluded from the analysis due to missing data for three of the four periods as a result of the inability to collect blood samples through the IV catheter due to a repeatedly clogged catheter line.

Seventy-six individuals attended a study information meeting, sixty-four of which were screened for eligibility. Of the 58 individuals found eligible, 36 were enrolled. The CONSORT diagram for the study is found in Figure 1.

Thirty-six subjects were enrolled in this study. At baseline, subjects had the following characteristics: age 58.7 years, weight 88.6 kg, body mass index 30.8 kg/m^2^, fasting glucose 94.2 mg/dL, fasting insulin 80.0 pmol/L, fasting triglycerides 122.1 mg/dL, systolic blood pressure 133.3 mm Hg, diastolic blood pressure 79.7 mm Hg (Table 1). Six subjects had fasting plasma glucose at or above 100 mg/dL, with the maximum value being 115 mg/dL; diabetics were excluded but pre-diabetics were not excluded. Thirty-three subjects completed all four diet periods, while three subjects missed one diet period. One subject completed all four diet periods, but due to repeated clogging of the IV catheter line, blood samples were not successfully collected during three of the four treatment periods, thus, the subject’s data were excluded. Samples and data from 35 of the 36 subjects were analyzed. For four of the 35 subjects, data from one treatment period were excluded due to extremely high values for glucose and or insulin; in two cases, catheter problems were noted, in two other cases, no problems were noted but data were 3 standard deviations above the group mean.

The daily intake of anthocyanins from whole berries during the controlled feeding ranged from 109 mg/day to 218 mg/day, with a mean intake of 143.5 mg/day. When berry juice was consumed daily, the daily anthocyanin intake ranged from 77 mg/day to 155 mg/day, with a mean intake of 101 mg/day. Cyanidin 3-glucoside was the predominant anthocyanin, at a level of 14 mg/100 g of mixed berries and 6.6 mg/100 g of pressed juice. The whole berries also provided a mean intake of 15 mg/day flavonols, 3 mg/day flavan-3-ols, and 38 mg/day phenolic acids, while the berry juice provided a mean intake of 6 mg/day flavonols, less than 1 mg/day flavan-3-ols, and 6 mg/day phenolic acids. The whole berry portions provided for the meal based oral glucose tolerance tests provided 82 mg anthocyanins, 9 mg flavonols, 2 mg flavan-3-ols, and 22 mg phenolic acids, while the juice portions provided for the meal based oral glucose tolerance tests provided 58 mg anthocyanins, 4 mg flavonols, less than 1 mg flavan-3-ols, and 4 mg phenolic acids. The intake of phenolic compounds was greater during the whole berry treatment period compared to the berry juice treatment period because the whole berries and the juice doses were chosen to be matched for sugar (and calories), which are more readily extracted during pressing that the phenolic compounds. The gelatin control foods were devoid of phenolic compounds. A description of the carbohydrate and polyphenol content of treatment foods is found in Table 2 and the polyphenol composition of the mixed berry and berry juice treatments is found in Table 3.

**Figure 2:**
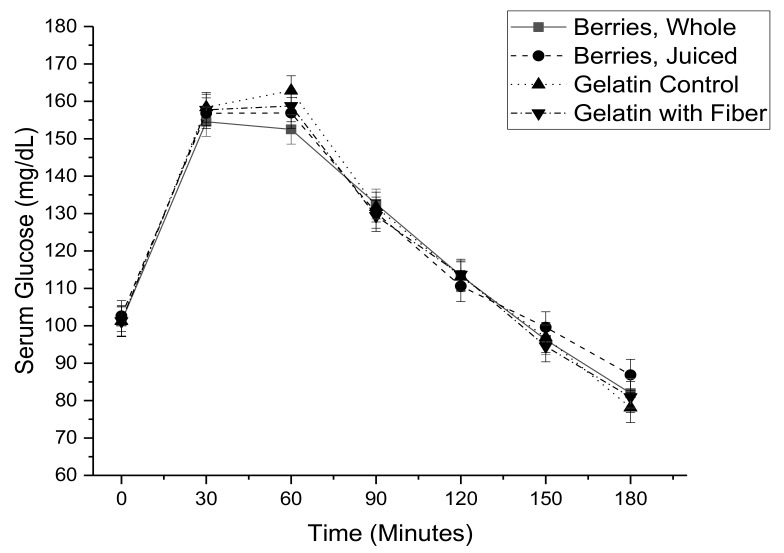
Serum glucose as a function of time during a meal-based glucose tolerance test with co-consumption of either whole mixed berries, pressed mixed berry juice, a sugar matched gelatin control, or a sugar matched fiber-enriched gelatin control.

**Figure 3:**
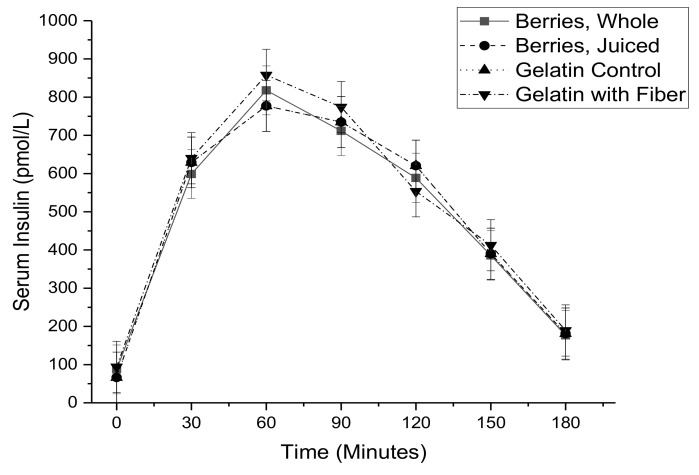
Serum insulin as a function of time during a meal-based glucose tolerance test with co-consumption of either whole mixed berries, pressed mixed berry juice, a sugar matched gelatin control, or a sugar matched fiber-enriched gelatin control.

**Figure 4:**
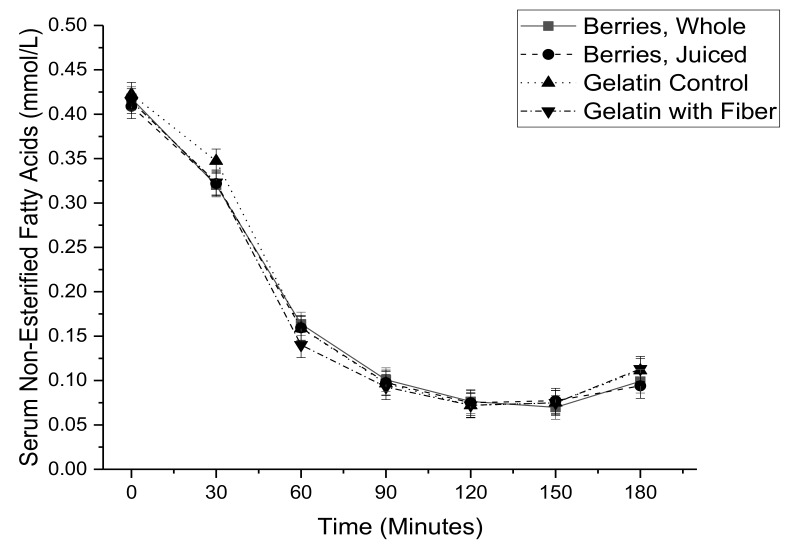
Serum non-esterified fatty acids as a function of time during a meal-based glucose tolerance test with co-consumption of either whole mixed berries, pressed mixed berry juice, a sugar matched gelatin control, or a sugar matched fiber-enriched gelatin control.

Serum glucose (Figure 2) and serum insulin (Figure 3) were elevated by the meal based oral glucose tolerance test, while serum non-esterified fatty acids (Figure 4) were decreased. There were no significant differences related to treatment for individual time points. These concentration by time data were used to calculate the area under the concentration time curve.

Baseline glucose, insulin, and non-esterified fatty acids were not affected by treatment. Further, neither glucose incremental AUC nor insulin incremental AUC were significantly affected by treatment. Similarly, non-esterified fatty acid AUC was not affected by treatment. These results are shown in Table 4.

As a secondary analysis, data from the berry treatments (whole berries plus pressed berry juice) were combined and compared to combined data from the non-berry treatments (sugar matched gelatin plus sugar matched fiber-enriched gelatin). The combined data demonstrated a significant reduction in serum insulin AUC (*p* = 0.023) when the berry treatments were consumed compared to the gelatin control foods. Further, the difference for serum glucose AUC was near statistical significance (*p* = 0.074) for a lowering when berry treatments were consumed. There was no effect on AUC for non-esterified fatty acids. These data are shown in Table 5.

## 4. Discussion

The purpose of this study was to investigate the potential glucoregulatory effects of mixed berries in adults consuming a high fat diet under very highly controlled conditions. Previous studies of glucoregulatory effects of berries have been mostly positive, although the results have been mixed. Possible reasons for mixed results include the lack of a controlled diet and differences in the berry products provided, which are usually extracts or powders.

Despite the very careful development of the control foods and the investigator-provided diet controlling energy, macronutrients, micronutrients, and polyphenols preceding each glucose tolerance test, there were no differences among the individual treatments for serum insulin, serum glucose, or serum non-esterified fatty acid response despite the fact that some previous studies with berry preparations have demonstrated beneficial effects on these measures. Possible reasons for such a finding include higher than average variability amongst the study population, a less effective berry preparation, or an insufficient dose. Regarding population variability, there is no reason to suspect this population would have unusually large variability in response; the study population was recruited from healthy adults living in the Baltimore/DC vicinity and was as large or larger than study populations enrolled in most other similar studies. Regarding berry form, previous studies demonstrating glucoregulatory benefit have been conducted with whole berries as well as berry extracts or powders. An acute study of type 2 diabetics demonstrated significant reductions in insulin and glucose AUC after ingestion of 470 mg of a bilberry extract [15] and similar findings were reported in a separate acute study of overweight men and women fed 600 mg anthocyanins from a black currant extract in a single meal [8]. These findings are supported by follow-up work that suggested one mode of action of anthocyanins on blood sugar is the decreased digestion and/or absorption of carbohydrates in the gut [5]. Studies that utilize freeze-dried powder forms of strawberries or blueberries also report benefits on insulin sensitivity when berry powders are fed for several weeks [6,17,41]. However, null findings on glucoregulation have also been reported in studies utilizing berry powders or extracts [23,24,25,42,43,44]. Some studies utilizing whole berry interventions, as opposed to powders or extracts, have reported positive findings in acute feeding settings with healthy subjects utilizing meal-based glucose tolerance tests [7,9,10]. Finally, it is possible that some berries are more effective than others, and the inclusion of mixed berries in this study served to dilute the effectiveness of the most potent berries. An acute feeding study in overweight or obese subjects demonstrates the potential for differences in the type of berry fed, as an improved glycemic profile and reduced insulin response was noted with sea buckthorn berries, but not strawberries [45], although freeze-dried strawberry powder conveyed beneficial effects for glucoregulation in other trials [17,41]. A recent 8-week intervention study in subjects at risk for type 2 diabetes reports positive effects of a bilberry intervention, but not a combination strawberry-raspberry-cloudberry intervention, on both fasting glucose and improvements in the disposition index following an OGTT [46]. Anthocyanin dose may influence efficacy as well. For the study of bilberry vs. strawberry-raspberry-cloudberry, the bilberry intervention was much higher in anthocyanins than the strawberry-raspberry-cloudberry intervention; specifically, the bilberry preparation provided 1323 mg of anthocyanins per day compared to 71 mg of anthocyanins per day for the mixed berry intervention [46]. We have previously reported the significant effects of blackberry feeding on insulin and non-esterified fatty acid plasma concentrations in overweight and obese subjects fed a controlled high-fat diet for 7 days. In that study, subjects consumed 361 mg/day of anthocyanins as cyanidin 3-glucoside from whole blackberries. For the current study, which had a similar design, subjects consumed on average 143 mg/day of mixed anthocyanins during the whole berry intervention [19]. However, studies with doses as low as a single bolus of anthocyanins at 81.6 mg from strawberry powder [16] or 70 mg from maqui berry extract [43] reduced post-prandial insulin and glucose response, respectively. Regarding study power, the number of subjects enrolled in this study met or surpassed most other studies as well, so it is unlikely that statistical power was limited in comparison.

When the berry treatments were combined and compared to the combined gelatin treatments, the berry treatments showed improvement in serum insulin and a value close to statistical significance for improvement of serum glucose. This result generally supports the role of berries in improving glucoregulation and potentially reducing risk for diabetes, in accord with previous human intervention studies demonstrating beneficial effects of berries on insulin sensitivity and glucoregulation [5,6,7,8,9,10,11,12,13,14,15,16,17,18,19]. Perhaps the effectiveness of berry preparations on glucoregulatory parameters is determined by a combination of berry type, source preparation, and bioavailability.

The strengths of this study include the very tightly controlled diet prior to the glucose tolerance test and the very carefully designed control foods to provide the same types and amounts of sugar and fiber. The weakness of this study is the combination of pre-feeding berries prior to the glucose tolerance test and with the glucose tolerance test. Feeding berries both before and during the glucose tolerance test prevents determination of whether long-term feeding or acute feeding is important for harnessing the beneficial effects of berries. However, evidence from previous studies suggests that both shorter-term [7,8,9,10,16,17] and longer-term [6,12,20] effects exist.

## 5. Conclusions

These results suggest the potential for anthocyanin-rich mixed berry preparations to improve post-prandial insulin and possibly glucose response.

## Figures and Tables

**Table 1:** Baseline characteristics of study subjects.

	Mean ± SE, *n* = 36 (18M/18F)
Age (years)	58.7 ± 1.9
Weight (kg)	88.6 ± 2.6
Body mass index (kg/m^2^)	30.8 ± 0.6
Fasting glucose (mg/dL)	94.2 ± 1.3
Fasting insulin (pmol/L)	80.0 ± 7.4
Fasting triglycerides (mg/dL)	122.1 ± 7.7
Systolic blood pressure (mm Hg)	133.3 ± 2.4
Diastolic blood pressure (mm Hg)	79.7 ± 2.0

**Table 2:** Range and mean dose, carbohydrate content, and polyphenol content in treatment foods consumed daily as part of a controlled diet ^1^.

	Gelatin Control	Whole Berries	Berry Juice	Gelatin + Fiber
Daily dose (g)	400–800 (523)	400–800 (523)	384–768 (502)	400–800 (523)
Total sugar (g)	28.8–57.6 (37.6)	28.8–57.6 (37.6)	28.6–57.3 (37.4)	28.8-57.6 (37.6)
Glucose (g)	15.0–30.0 (19.6)	15.0–30.0 (19.6)	15.0–30.0 (19.6)	15.0–30.0 (19.6)
Fructose (g)	13.8–27.6 (18.0)	13.8–27.6 (18.0)	13.6–27.3 (17.8)	13.8–27.6 (18.0)
Soluble fiber (g)	0	0	0	0
Insoluble fiber (g)	0	7.2–14.5 (9.5)	0	7.2–14.5 (9.5)
Anthocyanins (mg) ^2^	0	109.0–218.0 (143.5)	77.3–154.6 (101.1)	0
Flavonols (mg) ^2^	0	11.7–23.4 (15.3)	4.9–9.8 (6.4)	0
Flavan-3-ols (mg) ^2^	0	2.2–4.5 (2.9)	0.5–0.9 ((0.7)	0
Phenolic acids (mg) ^2^	0	29.2–58.4 (38.2)	4.7–9.4 (6.1)	0

^1^ Treatment doses were scaled according to total energy intake for each subject; values are dose range followed by mean in parentheses; ^2^ Detailed polyphenol composition for whole berries and berry juice is shown in Table 3.

**Table 3:** Polyphenol composition of mixed berries and juice treatments ^1^.

		mg per 100 g Fresh Weight		Whole Berries		Pressed Berry Juice	
		Mixed Berries, Whole	Pressed Juice	Polyphenols in mg per 400 g ^2^	Polyphenols in mg per 800 g ^3^	Polyphenols in mg per 384 g ^4^	Polyphenols in mg per 768 g ^5^
**Anthocyanins**	Cyanidin-3-Gal	1.70 ± 0.08	3.19 ± 0.05	6.81	13.6	12.3	24.5
	Cyanidin-3-Glu	14.05 ± 0.96	6.57 ± 0.20	56.2	112.4	25.2	50.4
	Cyanidin-3-Ara	0.906 ± 0.168	2.01 ± 0.05	3.63	7.25	7.70	15.4
	Delphinidin-3-Gal	0.874 ± 0.103	0.101 ± 0.002	3.50	6.99	0.39	0.78
	Delphinidin-3-Glu	ND	trace	ND	ND	ND	ND
	Delphinidin-3-Ara	0.830 ± 0.081	0.053 ± 0.002	3.32	6.64	0.21	0.41
	Malvidin-3-Gal	2.54 ± 0.15	1.05 ± 0.06	10.2	20.3	4.04	8.07
	Malvidin-3-Glu	0.500 ± 0.060	0.012 ± 0.003	2.00	4.00	0.05	0.09
	Malvidin-3-Ara	3.05 ± 0.17	0.805 ± 0.017	12.2	24.4	3.09	6.18
	Peonidin-3-Gal	0.793 ± 0.102	3.43 ± 0.10	3.17	6.34	13.2	26.4
	Peonidin-3-Glu	0.070 ± 0.012	0.17 ± 0.02	0.28	0.56	0.64	1.27
	Peonidin-3-Ara	0.588 ± 0.075	2.53 ± 0.06	2.35	4.71	9.72	19.44
	Petunidin-3-Gal	0.920 ± 0.039	0.159 ± 0.003	3.68	7.36	0.61	1.22
	Petunidin-3-Glu	0.340 ± 0.011	0.016 ± 0.004	1.36	2.72	0.06	0.12
	Petunidin-3-Ara	0.076 ± 0.066	0.057 ± 0.002	0.30	0.60	0.22	0.44
Total	Anthocyanins	27.2 ± 1.9	20.1 ± 0.4	109.0	218.0	77.3	154.6
**Phenolic acids**	Chlorogenic acid	7.09 ± 0.27	1.08 ± 0.02	28.4	56.7	4.14	8.28
	Caffeic acid	0.211 ± 0.013	0.032 ± 0.002	0.84	1.69	0.12	0.24
	Gallic acid	ND	0.116 ± 0.004	ND	ND	0.44	0.89
Total	Phenolic acids	7.30 ± 0.28	1.23 ± 0.02	29.2	58.4	4.71	9.42
**Flavonols**	Quercetin-3-Gal	2.01 ± 0.64	1.00 ± 0.01	8.06	16.1	3.84	7.68
	Quercetin-3-Glu	0.448 ± 0.001	0.088 ± 0.001	1.79	3.58	0.34	0.67
	Kaempferol-3-Glu	0.466 ± 0.042	0.186 ± 0.003	1.86	3.73	0.71	1.43
Total	Flavonols	2.93 ± 0.67	1.27 ± 0.01	11.7	23.4	4.89	9.78
**Flavan-3-ols**	Catechin	0.226 ± 0.026	0.079 ± 0.003	0.90	1.81	0.30	0.60
	Epicatechin	0.330 ± 0.016	0.039 ± 0.001	1.32	2.64	0.15	0.30
Total	Flavan-3-ols	0.556 ± 0.041	0.118 ± 0.004	2.22	4.45	0.45	0.90

^1^ Values are mean ± standard deviation; ND indicates not detected; ^2^ Daily whole berry dose for the lowest calorie level; ^3^ Daily whole berry dose for the highest calorie level; ^4^ Daily pressed berry juice dose for the lowest calorie level; ^5^ Daily pressed berry juice dose for the highest calorie level.

**Table 4:** Glucose, insulin, and non-esterified fatty acid response as area under the serum concentration time curve after the meal-based oral glucose tolerance test.

	Gelatin Control ^1^	Whole Berries ^1^	Berry Juice ^1^	Gelatin + Fiber ^1^	*p*-Value ^2^
Glucose iAUC ^3^ (ln mg·min/dL)	3.38 ± 0.05	3.32 ± 0.05	3.34 ± 0.05	3.31 ± 0.05	0.19
Insulin iAUC ^4^ (ln pmol·min/L)	11.40 ± 0.08	11.31 ± 0.08	11.32 ± 0.08	11.39 ± 0.8	0.13
NEFA AUC ^5^ (ln mmol·min/L)	9.03 ± 0.08	8.91 ± 0.08	8.90 ± 0.08	8.96 ± 0.08	0.65

^1^ Values are LS Mean ± SEM; ^2^ *p* value for treatment effect; ^3^ Glucose iAUC is incremental area under the serum concentration time curve for glucose; ^4^ Insulin iAUC is incremental area under the serum concentration time curve for insulin; ^5^ NEFA AUC is area under the serum concentration time curve for non-esterified fatty acids.

**Table 5:** Difference in glucose, insulin, and non-esterified fatty acid area under the curve for combined gelatin treatments vs. combined berry treatments.

	Difference between Gelatin Treatments vs. Berry Treatments ^1^	*p*-Value ^2^
Glucose iAUC (ln mg·min/dL)	0.169 ± 0.093	0.074
Insulin iAUC (ln pmol·min/mL)	0.153 ± 0.066	0.023
NEFA AUC (ln mmol·min/L)	0.028 ± 0.062	0.648

^1^ Values are LS Mean ± SEM, positive difference indicates lower value for berry treatments; ^2^ *p* value for treatment effect.
